# Account of Diseases in an Eastern District of London

**Published:** 1802-08-01

**Authors:** 


### ACCOUNT OF DISEASES IN AN EASTERN DISTRICT OF LONDON,

*From June* 20, *to July* 20, 1802.

ACUTE DISEASES.

| | |
|---|---|
| Febris Intermittens Tertiana | 4 |
| Scarlatina Anginoſa - - - | 4 |
| Pleuritis - - - - - - - | 3 |
| Eryſipelas - - - - - - | 1 |
| Rheumatiſmus Acutus - - | 5 |

CHRONIC DISEASES.

| | |
|---|---|
| Tuſſis - - - - - - - - | 12 |
| Dyſpnœa - - - - - - - | 7 |
| Tuſſis cum Dyſpnœa - - | 9 |
| Hæmoptyſis - - - - - | 2 |
| Hydrops Pectoris - - - | 2 |
| Anaſarca - - - - - - - | 3 |
| Pleurodyne - - - - - - | 4 |
| Epilepſia - - - - - - - | 2 |
| Cephalalgia - - - - - - | 7 |
| Paralyſis - - - - - - - | 2 |
| Syncope - - - - - - - | 2 |
| Hyſteria - - - - - - - | 4 |
| Gaſtrodynia - - - - - - | 7 |
| Diarrhœa - - - - - - - | 4 |
| Aſcites - - - - - - - | 3 |
| Hæmorrhois - - - - - - | 3 |
| Chloroſis - - - - - - - | 4 |
| Hernia - - - - - - - - | 2 |
| Scrophula - - - - - - - | 7 |
| Impetigo - - - - - - - | 4 |
| Rheumatiſmus Chronicus - | 12 |

PUERPERAL DISEASES.

| | |
|---|---|
| Abſceſſus Mammæ - - - | 2 |
| Menorrhagia Lochialis - - | 3 |
| Ephemera - - - - - - - | 4 |

INFANTILE DISEASES.

| | |
|---|---|
| Pertuſſis - - - - - - - | 4 |
| Ophthalmia - - - - - - | 3 |
| ———— Purulenta - - | 1 |
| Herpes - - - - - - - - | 3 |
| Tinea - - - - - - - - - | 2 |

With a ſtate of the weather ſo unuſual at this time of the year as that which we have lately experienced, and eſpecially with a temperature of the air continued for a conſiderable time ſo far below the point to which it generally riſes at the preſent ſeaſon, we might well expect that the human frame as well as the vegetable productions ſhould be materially affected: We have accordingly obſerved an unuſual prolongation of thoſe complaints which uſually accompany ſuch a ſtate of the air. Colds and coughs, pleuriſies and peripneumonies, rheumatic affections and pains in the face, have been very frequent. Hooping cough though, the number of patients labouring under it are fewer, and the ſymptoms attending it are leſs violent, is ſtill a troubleſome diſeaſe. Scarlatina anginoſa has prevailed to a conſiderable degree, and has in ſome inſtances proved fatal. In other inſtances it has been ſucceeded by anaſarcous ſwellings. This ſymptom does not in general excite any alarm, as it does in thoſe caſes where it may be conſidered as a primary diſeaſe, or as connected with the diſeaſed ſtate of ſome organ. It generally ſubſides in a little time after the uſual means have been employed.

*Observations*

